# STAT3 and IL-6 Contribute to Corticosteroid Resistance in an OVA and Ozone-induced Asthma Model with Neutrophil Infiltration

**DOI:** 10.3389/fmolb.2021.717962

**Published:** 2021-10-25

**Authors:** Yishu Xue, Yan Zhou, Wuping Bao, Qiang Fu, Huijuan Hao, Lei Han, Xue Zhang, Xue Tian, Min Zhang

**Affiliations:** Department of Respiratory and Critical Care Medicine, Shanghai General Hospital, Shanghai Jiao Tong University School of Medicine, Shanghai, China

**Keywords:** T2-low asthma, STAT3, IL-6, SOCS3, steroid resistance, ozone

## Abstract

Exposure to high levels of ozone contributes to insensitivity to glucocorticoids in asthma treatment, but the underlying mechanisms are not known. We built two asthma models: a “T2-high” asthma model was established by ovalbumin (OVA) sensitization/challenge and OVA sensitization/challenge combined with ozone exposure (OVA + ozone) was used to induce airway inflammation with increased numbers of neutrophils to simulate “T2-low” asthma. The expression of T-helper (Th)1/2/17-related cytokines was measured by cytokine antibody arrays. Bronchial provocation tests were carried out to evaluate the lung resistance of mice. Hematoxylin and eosin staining, periodic acid-Schiff staining, and immunohistochemical (IHC) analyses of alpha-smooth muscle actin were undertaken to observe morphology changes in lungs. The expression of glucocorticoid receptors (GRs) and phosphorylated-GR (p-GR) was measured by western blotting. *Nr3c1* mRNA was quantified by RT-qPCR. Protein expression of proinflammatory cytokines, signal transducer and activator of transcription 3 (STAT3), suppressor of cytokine signaling 3 (SOCS3), and CXCL1 was measured through ELISAs, western blotting, or IHC analyses. Resected lung tissue from seven asthma patients and 10 healthy controls undergoing thoracotomy for pulmonary nodules was evaluated by IHC analyses and ELISAs. In both asthma models, mucus hypersecretion, as well as inflammation, hyperresponsiveness, and remodeling of the airways, was present compared with the control group, whereas the OVA + ozone group showed severe neutrophil infiltration. The expression of Th17-related cytokines (interleukin (IL)-6, IL-17A, IL-21), GR protein, and CXCL1 increased in the OVA + ozone group, whereas the expression of p-GR decreased. Dexamethasone (Dex) could not totally reverse the expression of p-GR and histone deacetylase-2 in the OVA + ozone group. STAT3 expression increased in the OVA + ozone group and could not be completely reversed by Dex, and nor could IL-6 expression. A positive correlation between IL-6 or IL-17A and STAT3 and negative correlation between SOCS3 and STAT3 were shown, suggesting that the IL-6/STAT3 pathway may be involved in OVA + ozone–induced corticosteroid-resistant airway inflammation. In clinical samples, IL-17A expression in lung tissue was positively correlated with percent STAT3-positive area and negatively correlated with SOCS3 expression. The IL-6/STAT3 pathway may contribute to corticosteroid insensitivity in OVA + ozone–induced neutrophilic airway inflammation through regulation of Th17 cells and could provide new targets for individual treatment of corticosteroid resistance in asthma.

## Introduction

In 2009, Wenzel et al. (1999) defined two distinct inflammatory endotypes of asthma: T-helper type 2 (T2)-high and T2-low. They have remained the most well-recognized and described endotypes of asthma (Svenningsen S and Nair P, 2017).

T2-high asthma is characterized by eosinophilic airway inflammation. It presents with an immune response involving mainly T-helper type 2 (Th2)-related cytokines and, in general, is sensitive to corticosteroid therapy. T2-low asthma is usually characterized by neutrophilic airway inflammation and often exhibits an immune response mediated by interleukin (IL)-17 and Th17 cells (Chung, 2016).

Glucocorticoids are the main choice of asthma treatment due to their significant anti-inflammatory, immunosuppressive, and immunomodulatory effects (Alangari, 2014; Barnes, 2017). However, ≤10% of asthma patients do not respond to glucocorticoids even at high doses or in combination with oral corticosteroids (Chung, 2016). This absence of response makes asthma difficult to control and consumes considerable medical resources.

It has been revealed that proinflammatory cytokines can contribute to corticosteroid resistance in severe asthma and chronic obstructive pulmonary disease. Proinflammatory cytokines (e.g., IL-17A) together with allergens, pathogens, and cigarette smoke can modulate multiple signaling pathways, including nuclear factor, erythroid 2-like 2/histone deacetylase 2/c-Jun (Nrf2/HDAC/c-Jun), and a heightened glucocorticoid receptor (GR) ratio to induce corticosteroid insensitivity (Mei et al., 2019). Neutrophils are involved in corticosteroid insensitivity in asthma and may be regulated by Th17 cells (Chung, 2016; Panettieri, 2018).

An important pathway of initial differentiation of mouse Th17 cells requires IL-6 and signal transducer and activator of transcription 3 (STAT3). Murine and human Th17 cells express IL-17A, IL-17F, IL-21, IL-22, granulocyte-macrophage colony-stimulating factor, and IL-23R (Miossec and Kolls, 2012). STAT3 phosphorylation promotes the transformation of Th0 cells to Th17 cells (Cosmi et al., 2011). Suppressor of cytokine signaling 3 (SOCS3) is a negative regulator of Th17, which usually regulates Th17 cells through the interaction with STAT3 (Rottenberg and Carow, 2014).

STAT3–SOCS3 is an important pathway involved in inflammation modulation (Rottenberg and Carow, 2014). The role of STAT3 and SOCS3 was investigated in some models of allergic airway inflammation (Santana et al., 2019; Pinheiro et al., 2020), but the results were controversial. Their roles in asthma, especially T2-low asthma, are not clear.

Here, an asthma model with a mixed Th2/Th17 response was constructed by ovalbumin (OVA) sensitization/challenge combined with ozone exposure (OVA + ozone) to simulate some types of T2-low asthma in patients (Last et al., 2004; Zhang J.-h. et al., 2019; Zhang Y. et al., 2019) and show the features of corticosteroid resistance. The expression of total STAT3 was increased in both asthma models, and phosphorylated-STAT3 (p-STAT3) expression increased in the OVA + ozone group, whereas SOCS3 expression was reduced. The expression of STAT3 protein and IL-6 protein in lung tissue did not show an obvious response to corticosteroid intervention. We discovered higher STAT3 expression and lower SOCS3 expression in the lung tissue of asthma patients compared with that of healthy controls (HCs) and observed a correlation between STAT3 expression or SOCS3 expression and the expression of IL-17A protein. The findings from human tissue and mouse models suggested that, by regulating the expression of Th17 cytokines and related cytokines, STAT3 and IL-6 may contribute to corticosteroid insensitivity in an asthma model with a mixed Th2/Th17 response and may become a potential target for treatment of T2-low asthma.

## Materials and Methods

### Animals

Male C57BL/6 mice (specific-pathogen-free; 6 weeks) were purchased from SLRC Laboratory (Shanghai, China). They were housed in controlled conditions of temperature (21–25°C) and humidity (40–60%) and had free access to water and food (free from OVA). Mice were acclimatized for 7 days before experimentation and exposed to a 12 h light–dark cycle.

### Induction of Allergic Airway Inflammation and Ozone Exposure

Mice were divided randomly into four groups of eight. OVA sensitization was initiated on day 0 and day 7. OVA sensitization was achieved by intraperitoneal injection of 20 μg of OVA (grade V; Sigma-Aldrich, Saint Louis, MO, United States) dissolved in 0.2 ml of phosphate-buffered saline (PBS) emulsified in aluminum hydroxide (2 mg; Sigma-Aldrich) as an adjuvant. OVA challenge was undertaken on days 14, 16, 18, 20, and 22, with exposure to 5% aerosolized OVA (20 ml; grade II, Sigma-Aldrich) in a plastic box linked to an ultrasonic nebulizer (Clenny 2 Aerosol; Medel, San Polo di Torrile, Italy) for 30 min. Mice in the control group were sensitized and challenged with an identical volume (0.2 ml for sensitization and 20 ml for challenge) of PBS as a vehicle.

Mice were exposed to ozone (2 ppm) or air in a Perspex™ container 30 min after each OVA/PBS challenge on days 14, 16, 18, 20, and 22. Ozone was produced by an ozonizer (300 series; Aqua Medic, Bissendorf, Germany). Each exposure lasted for 2 h. The ozone concentration was controlled and adjusted by an OS-4 Ozone Switch (Ecosensor; KWJ Engineering, Newark, CA, United States) continuously. Mice in the control group were exposed to air during this time.

In the dexamethasone (Dex)-treated group (OVA + ozone + Dex), mice were injected (i.p.) with Dex (5 mg/kg; Solarbio, Beijing, China) each time before OVA challenge on days 14, 16, 18, 20, and 22.

### Airway Responsiveness

24 h after the last challenge, mice were anesthetized with Zoletil 50 (tiletamine hydrochloride and zolazepam hydrochloride, 25 mg/kg, Virbac S.A., France) and xylazine hydrochloride (10 mg/kg, Chang Sha Best Biological Technology Institute Co., Ltd., China) via IP injection. Mice under anesthesia were then ventilated (MiniVent™; Hugo Sachs Elektronik, March, Germany) at 180 breaths/min and a tidal volume of 210 μl in a whole-body plethysmograph with a pneumotachograph connected to a transducer (Electro-Medical Measurement Systems, Bordon, United Kingdom), as described previously (Bao et al., 2018). Pulmonary airway resistance (R_L_) was recorded during a 3 min period after increasing concentrations (4–256 mg/ml; 10 µl each time) of acetylcholine chloride (ACh; Sigma-Aldrich). R_L_ was expressed as percentage change from baseline R_L_ (measured following nebulization with PBS). The ACh concentration required to increase R_L_ by 100% from baseline (PC100) was calculated.

### Blood Collection, Bronchoalveolar Lavage, and Protein Isolation From Lung Tissue

After lung-function tests, mice were killed and blood was obtained from the right ventricle. Then, the trachea was exposed, and three aliquots of sterilized saline (0.6 ml each time) were instilled through a PE-60 tube. Bronchoalveolar lavage fluid (BALF) was retrieved (Bao et al., 2020), and the return volume was consistently ≥70% of the instilled volume. Samples of blood and BALF were centrifuged at 3,000 rpm for 10 min at 4°C, and the supernatants were used for measurements.

Lung tissues [for enzyme-linked immunosorbent assays (ELISAs)] were flushed with PBS before freezing. They were homogenized in lysis buffer containing protease and phosphatase inhibitors and then centrifuged for ELISAs, real-time reverse transcription–quantitative polymerase chain reaction (RT-qPCR), and western blotting.

### ELISAs

ELISAs of serum, BALF, and lung-tissue homogenates were conducted using commercial kits for IgE (Crystal Chem, Chicago, IL, United States), IL-6 and IL-17A (Anogen, Mississauga, Canada), IL-21, p-STAT3 (Tyr705), and total STAT3 (RayBiotech, Peachtree Corners, GA, United States), HDAC2 (EpiGentek, Farmingdale, NY, United States), and hypoxia inducible factor (HIF)-1α (Cell Biolabs, San Diego, CA, United States) according to the manufacturer’s instructions.

### Gene Expression

*Nr3c1* is the gene of GRs. Total RNA was isolated from lung tissue by TRIzol™ Reagent (Invitrogen, Carlsbad, CA, United States) and was translated into complementary DNA using the cDNA Reverse Transcription Kit (Applied Biosystems, Foster City, CA, United States) in a PTC-200 Peltier Thermal Cycler (MJ Research, Hercules, CA, United States). Real-time RT-qPCR was undertaken using the ViiA™ 7 Real-Time PCR System (Thermo Fisher, Waltham, MA, United States), as described previously (Bao et al., 2018). The mean fluorescence intensity of the internal reference gene (β-actin) and target gene (*Nr3c1*) was measured. The relative expression of target genes was calculated by the 2^−ΔΔCt^ method.

The primer sequences (forward and reverse, respectively) used for PCR were 5′-GAA GCA GAT GAG CCA TCA CTT-3′ and 5′-CGG TCC TTC TCT GAT AGT GGA-3′ for *Nr3c1* and 5′-CCT CTA TGC CAA CAC AGT-3′ and 5′-AGC CAC CAA TCC ACA CAG-3′ for β-actin.

### Measurement of Cytokine Levels in Serum

Levels of Th1-, Th2-, and Th17-related cytokines in the serum of mice were measured through cytokine antibody arrays (Mouse TH17 Array 1; RayBiotech), as described previously (Zhu et al., 2020), following the manufacturer’s instructions.

### Western Blotting

Total protein from lung tissues was extracted using RIPA lysis buffer (Beyotime Biotechnology, Shanghai, China). Extracted proteins were separated by sodium dodecyl sulfate–polyacrylamide gel electrophoresis and transferred to polyvinylidene difluoride (PVDF) membranes. The relative expression of proteins was determined using an ECL detection system (Li et al., 2021). The primary antibodies were those against GR (Cell Signaling Technology, Danvers, MA, United States), p-GR (Ser211; Cell Signaling Technology), SOCS3 (Thermo Scientific), and STAT3 (124H6; Cell Signaling Technology).

### Histology and Morphology

Mouse lungs were dissected out. The left lung was inflated by injecting 4% paraformaldehyde to provide 20 cm of water pressure. Then, it was immersed overnight in paraformaldehyde, embedded in paraffin, and sectioned for staining. Morphologic changes in the epithelia of lungs and airways were assessed by staining with hematoxylin and eosin (H&E) and periodic acid-Schiff (PAS).

Neutrophils, eosinophils, and lymphocytes located along lobar bronchi and segmental bronchi were counted (magnification = 400×) in a double-blinded manner by two investigators independently, as described previously (Bao et al., 2018). The mean width of the observed area was 100 μm. Peribronchiolar and perivascular inflammation observed in H&E-stained lung slices was scored from 0 to 3, as described previously (Bao et al., 2018).

Blue/purple-stained cells were identified as being PAS-positive. The percent PAS-positive area in the total epithelial area was calculated using ImageJ (National Institutes of Health, Bethesda, MD, United States).

### Immunohistochemical Analyses

IHC staining for α-smooth muscle actin (α-SMA), STAT3, and SOCS3 in lung-tissue sections with mouse α-SMA antibody (Proteintech, Chicago, IL, United States), SOCS3 antibody (Thermo Scientific), STAT3 antibody (124H6; Cell Signaling Technology), chemokine (C-X-C motif) ligand 1 (CXCL1), chemokine (C-X-C motif) receptor 2 (CXCR2) antibody (Proteintech, Chicago, IL, United States), and CXCR2 antibody (Servicebio, Wuhan, China) was done, as described previously (Zhang et al., 2018). The area of positive expression was identified in a double-blinded and independent fashion by two investigators using ImageJ.

### Patient Cohort

#### Inclusion Criteria

The inclusion criteria were patients 1) aged 16–75 years; 2) who accepted pneumectomy for lung nodules; 3) who completed spirometry before surgery; 4) had percent forced expiratory volume in one second (FEV_1_%) ≥80% predicted; 5) with FEV_1_/forced vital capacity (FVC) > 0.7 after salbutamol administration; and 6) with the diameter of pulmonary nodules <3 cm.

#### Exclusion Criteria

The exclusion criteria were patients 1) who had suffered a respiratory infection in the 8 weeks before screening; 2) who were pregnant; 3) who had concomitant systemic respiratory disease (including chronic obstructive pulmonary disease); and 4) who had other significant medical problems as determined by the principal investigator.

#### Grouping

Individuals with a history of asthma, confirmed to have variable symptoms (chronic recurrent wheezing, dyspnea, chest tightness, and/or cough) and variable airflow restriction (positive bronchodilation test or bronchial provocation test), were classified as the “asthma” group. Patients with no symptoms or other pulmonary diseases were classified as the “control” group.

### Statistical Analyses

Statistical analyses and graph creation were undertaken using Prism 8.0 (GraphPad, San Diego, CA, United States). However, the Spearman correlation coefficient matrix and Spearman rank correlation tests were undertaken using R 3.6.1 (R Institute for Statistical Computing, Vienna, Austria). Data are expressed as mean ± SD unless indicated otherwise. The Kruskal–Wallis one-way analysis of variance with Bonferroni’s *post hoc* test (for equal variance) or Dunnett’s T3 *post hoc* test (for unequal variance) was used to evaluate differences in variances between multiple groups. A post-test Mann–Whitney analysis was conducted to evaluate differences in variances between two groups. *p* < 0.05 was considered significant.

## Results

### Expression of Th17-Related Cytokines Increased in the OVA Group, but Expression of Th2-Related Cytokines Increased in the OVA + Ozone Group

**FIGURE 1:**
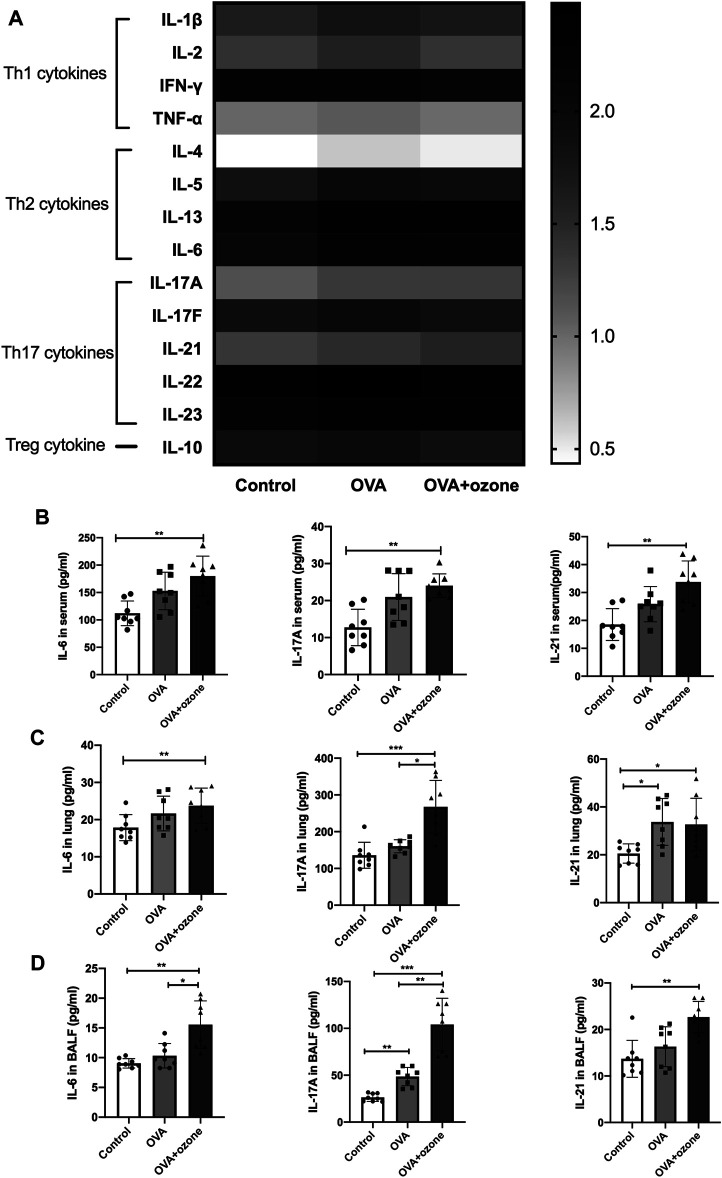
Expression of Th1-, Th2-, and Th17-related cytokines in the serum of mice and expression of Th17-related cytokines in the lung tissue and BALF of mice. The expression of cytokines in serum was measured by cytokine antibody arrays. The expression of Th17-related cytokines in BALF and lung tissue was measured through ELISAs. **(A)** Expression of different types of cytokines in serum (statistical analysis of cytokines is shown in Supplementary Figure S1). **(B)** Expression of Th17-related cytokines in serum. **(C)** Expression of Th17-related cytokines in lung tissue. **(D)** Expression of Th17-related cytokines in BALF. To reduce the difference in expression among different cytokines, the values in panel **(A)** are shown in logarithmic form, whereas the original values of measured protein concentration are used for the remainder of the plates. *p < 0.05; **p < 0.01; ***p < 0.001.

Cytokine antibody arrays were used to measure the expression of Th1-, Th2-, and Th17-related cytokines in the serum of each group (Figure 1A). The expression of Th1-related cytokines (IFN-γ, IL-2, and TNF-α) showed no significant difference among the two asthma models and control group. The expression of Th2-related cytokines (IL-4, IL-5, and IL-13) increased in the OVA group compared with the control group. IL-13 increased in the OVA + ozone group as well (Supplementary Figure S2). The expression of IL-10 (a cytokine associated with T regulatory cells) in mouse serum was higher in the OVA group. The expression of Th17-related cytokines (IL-17A and IL-21) was significantly higher in the serum of mice in the OVA + ozone group than that in the control group (Figure 1B). IL-6 expression was also increased in the OVA + ozone group. Although often classified as a Th2-related cytokine, IL-6 has an important role in a pathway of the initial differentiation of mouse Th17 cells (Miossec and Kolls, 2012).

Subsequently, the expression of Th17-related cytokines was measured in the BALF and lung-tissue homogenates of mice. Protein expression of IL-6, IL-17A, and IL-21 in the lungs of mice in the OVA + ozone group was significantly higher than that in the control group (Figure 1C). Protein expression of Th17-related cytokines in BALF was similar to that in serum. The expression of IL-6, IL-17A, and IL-21 in the lungs of mice in the OVA + ozone group was significantly higher than that in the control group. The expression of IL-6 and IL-17A in the lungs of mice in the OVA + ozone group was significantly higher than that in the OVA group (Figure 1D). These data suggested that Th17-related cytokines, rather than Th1- or Th2-related cytokines, may have important roles in our OVA + ozone mouse model.

### The OVA + Ozone Group Showed Severe Airway Inflammation, Hypersecretion, and Airway Hyperresponsiveness Compared With Those in the OVA Group

**FIGURE 2:**
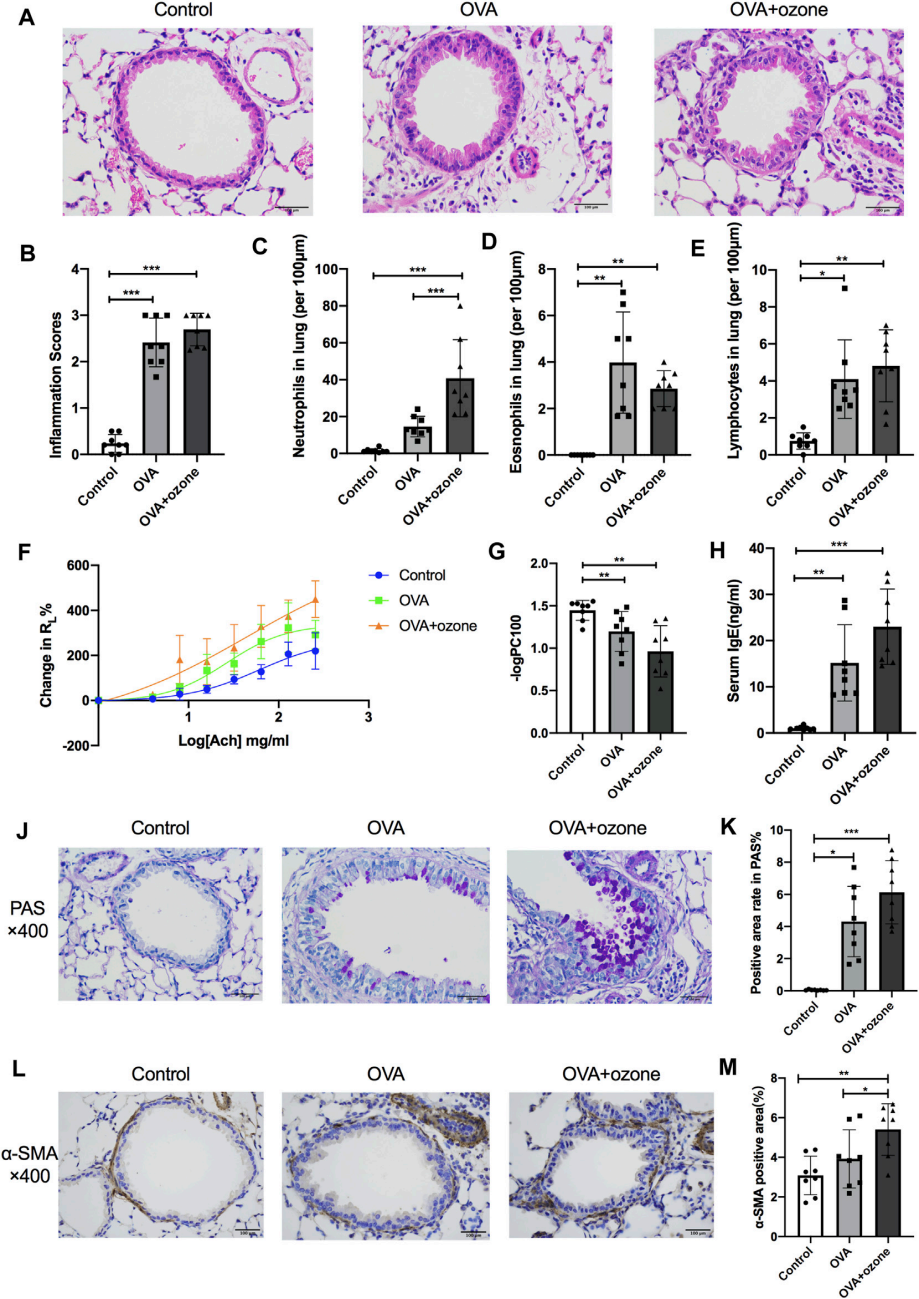
Mucus secretion, inflammation, responsiveness, and remodeling of the airways and IgE level of serum in T2-high and T2-low asthma models. Airway inflammation was evaluated through H&E staining. The IgE level in serum was analyzed by ELISAs. R_L_ (to acetylcholine chloride) was measured by a commercial setup from Electro-Medical Measurement Systems. **(A)** Representative photomicrographs of the lung with inflammatory-cell infiltration and hyperemia in hematoxylin and eosin–stained sections. **(B)** Airway inflammation scores. **(C)** Density of neutrophil infiltration. **(D)** Density of eosinophil infiltration. **(E)** Density of lymphocyte infiltration. **(F)** Mean percent increase in R_L_ in response to increasing concentrations of ACh. **(G)** –logPC100 (the ACh concentration required to increase R_L_ by 100% from baseline). **(H)** IgE level in serum. **(J)** Representative photomicrographs of the lung with airway mucus production and goblet-cell hyperplasia. **(K)** PAS-positive area. **(L)** Representative photomicrographs of immunohistochemistry of α-SMA in lung-tissue slices. **(M)** Percent SMA-positive area around airways. ACh, acetylcholine chloride; R_L_, lung resistance; α-SMA, α-smooth muscle actin. *p < 0.05; **p < 0.01; ***p < 0.001. Scale bar = 100 μm.

Compared with the control group, the OVA group and OVA + ozone group showed significant airway inflammation (as reflected in airway thickening) as well as infiltration of inflammatory cells (e.g., eosinophils, neutrophils, and lymphocytes) around the trachea and blood vessels (Figures 2A–E). The OVA + ozone group showed more neutrophil infiltration than that in the OVA group (Figure 2C). Airway hyperresponsiveness (Figures 2F,G) and increased levels of IgE in serum (Figure 2H) were observed in both endotypes of asthma models. The ozone group showed neutrophilic infiltration (Supplementary Figures S4A,B,D) and airway hyperactivity (Supplementary Figures S4E,F) compared to the control group, while its eosinophilic infiltration around the trachea and blood vessels showed no statistical differences from that in the control group (Supplementary Figure S4C).

Airway hypersecretion was observed in mice of both asthma-model groups (Figure 2J). The percent positive PAS-stained area demonstrated that increased mucus secretion occurred in the OVA group and OVA + ozone group compared with that in the control group (Figure 2K).

Airway smooth muscle (ASM) mass (as indicated by the amount of α-SMA around the airways) increased in lung slices from mice treated with OVA + ozone compared with that in the control group (Figures 2L,M). The OVA group had a slightly (but not significantly) thicker ASM mass than control mice. The ASM mass in the lung slices of mice of the OVA + ozone group increased more than that of the OVA group (Figure 2M), which indicated severe airway remodeling in the OVA + ozone group.

### The OVA + Ozone Group Showed Features of Corticosteroid Resistance

**FIGURE 3:**
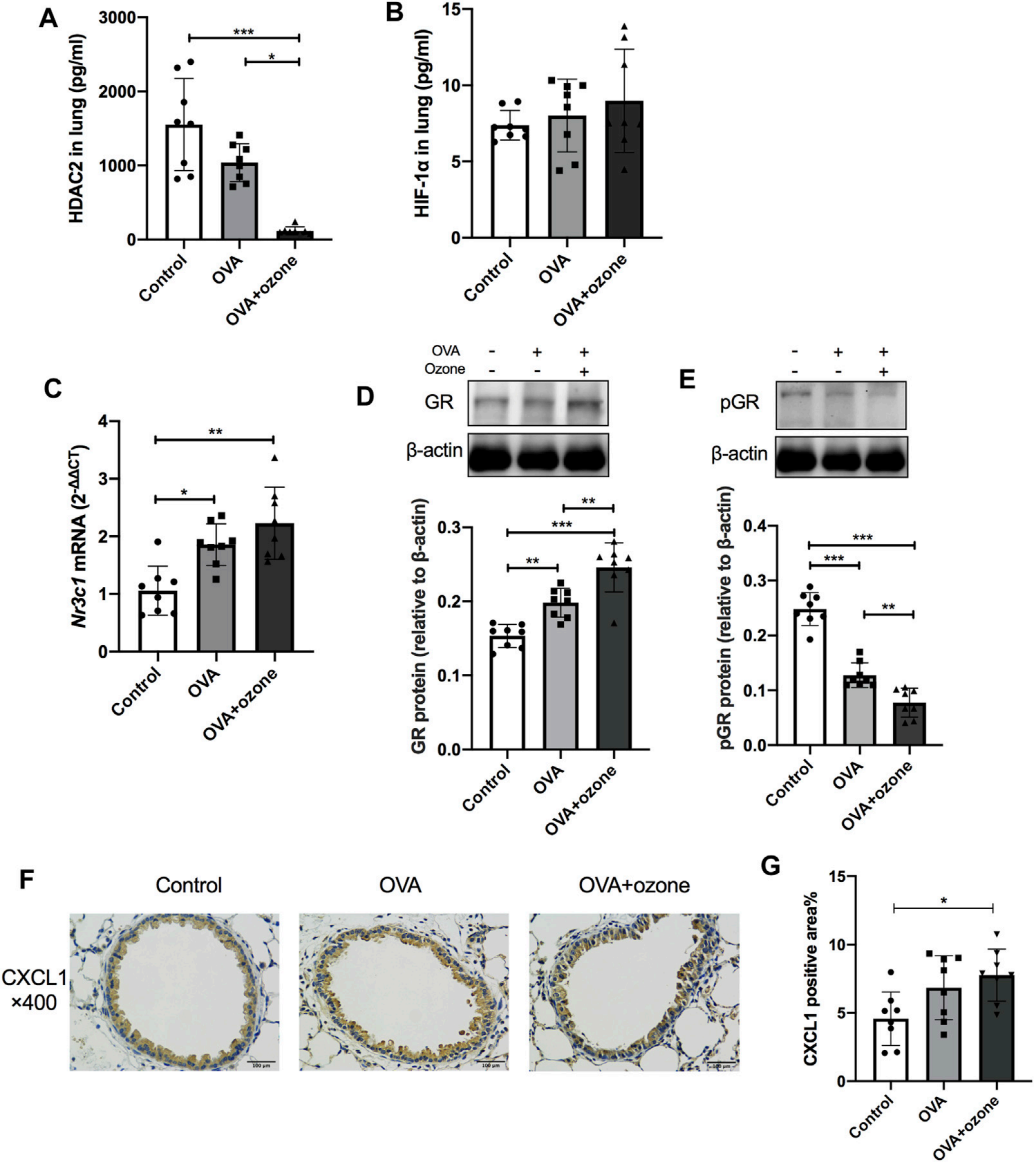
Expression of cytokines and receptors related to corticosteroid resistance in the lung tissue of mice suffering from asthma. The expression of HDAC2 and HIF-1α in the lung tissue of mice was measured by ELISAs. *Nr3c1* (gene of GRs) mRNA was detected by RT-qPCR. The expression of GR protein and p-GR protein was measured by western blotting. **(A)** HDAC2 expression in lung tissue. **(B)** HIF-1α expression in lung tissue. **(C)** Expression of *Nr3c1* mRNA in lung tissue. **(D)** Expression of GR protein in lung tissue. **(E)** Expression of p-GR protein in lung tissue. **(F)** Representative photomicrographs of immunohistochemistry of CXCL1 in lung-tissue slices. **(G)** Percent CXCL1-positive area around airways. CXCL1, chemokine (C-X-C motif) ligand 1; GR, glucocorticoid receptor; HDAC2, histone deacetylase 2; p-GR, phosphorylated-GR. *p < 0.05; **p < 0.01; ***p < 0.001.

As mentioned above, the number of neutrophils and expression of Th17-related cytokines were increased in the OVA + ozone group, which could lead to corticosteroid resistance. Protein expression of HDAC2 (which is related to corticosteroid resistance) decreased in the lung tissues of mice in the OVA + ozone group (Figure 3A). As a model involving neutrophil infiltration induced by oxidative stress, the OVA + ozone group showed a trend of increased HIF-1α expression, but there was no significant difference between the OVA + ozone group and the control group (Figure 3B). Compared with that in the control group, the expression of *Nr3c1* mRNA in the lung tissue of mice in the OVA + ozone group increased (Figure 3C). Correspondingly, the expression of GR protein increased significantly in the OVA + ozone group (Figure 3D). However, the expression of p-GR in the lung tissue of the OVA + ozone group decreased more than that in the OVA group (Figure 3E). GR phosphorylation may have been involved in corticosteroid resistance observed in the OVA + ozone group.

CXCL1 significantly increased in the lung of the OVA + ozone group compared to the control group (Figures 3F,G), while the positive area percentage of CXCR2 showed no statistical difference among the three groups (Supplementary Figure S2).

### STAT3 Expression Increased and SOCS3 Expression Decreased in the OVA + Ozone Group

**FIGURE 4:**
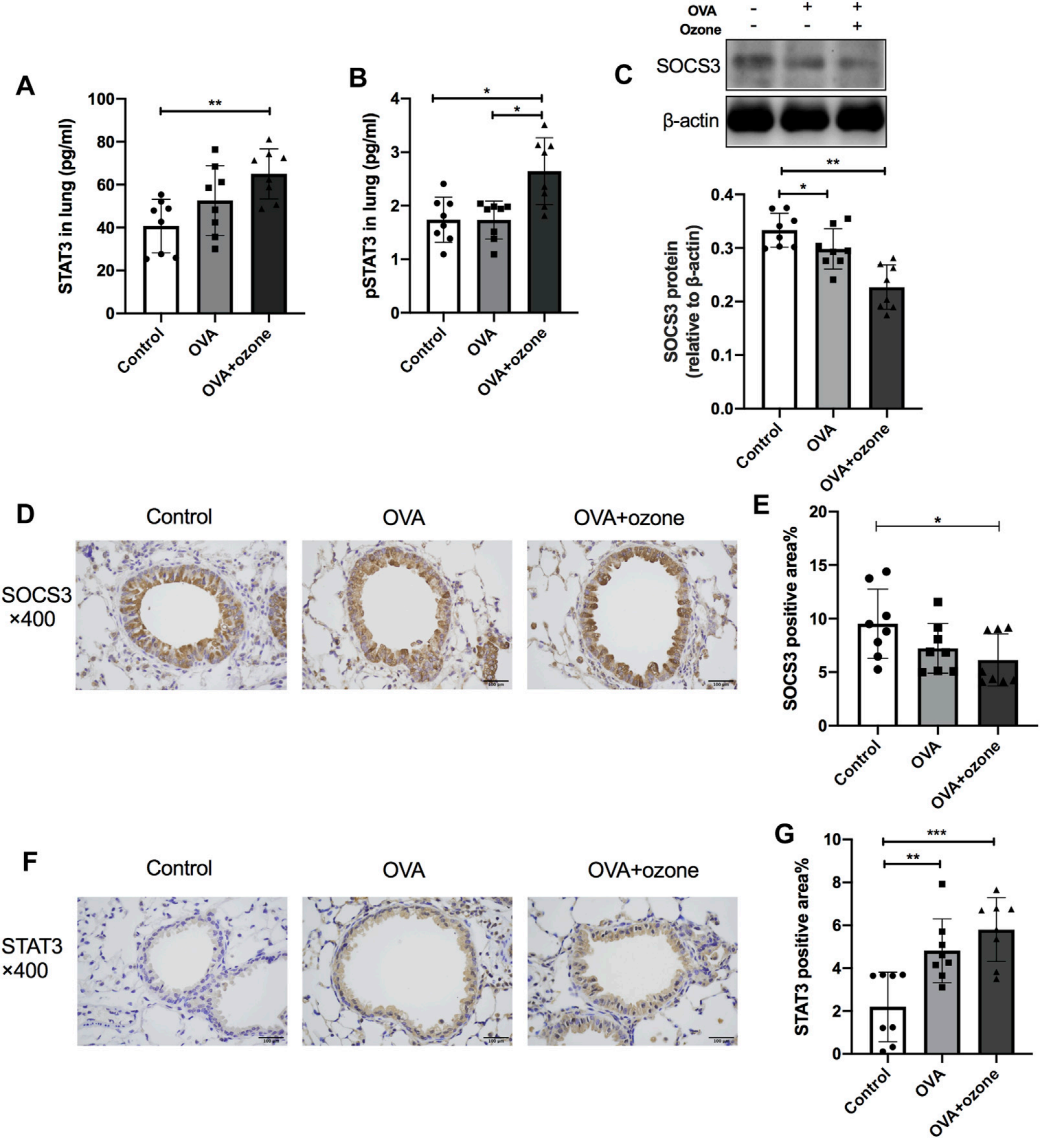
Expression of STAT3 and SOCS3 in the lung tissue of mice suffering from asthma. The expression of STAT3 and p-STAT3 in the lung tissue of mice was measured by ELISAs. The expression of SOCS3 protein in the lung tissue of mice was measured by western blotting, and protein expression of STAT3 and SOCS3 in the lung tissue of mice was evaluated by immunohistochemistry. **(A)** Expression of STAT3 protein in lung tissue. **(B)** Expression of p-STAT3 protein in lung tissue. **(C)** Expression of SOCS3 protein in lung tissue. **(D)** Representative photomicrographs of immunohistochemistry of SOCS3 in lung-tissue slices. **(E)** Percent SOCS3-positive area around airways. **(F)** Representative photomicrographs of immunohistochemistry of STAT3 in lung-tissue slices. **(G)** Percent STAT3-positive area around airways. SOCS3, suppressor of cytokine signaling 3; STAT3, signal transducer and activator of transcription 3; p-STAT3, phosphorylated-STAT3. *p < 0.05; **p < 0.01; ***p < 0.001. Scale bar = 100 μm.

The expression of total STAT3 and p-STAT3 (phosphorylated at Y705) in the lung tissues of mice in the OVA + ozone group increased, and the expression of p-STAT3 showed a significant increase compared with that in the OVA group (Figures 4A,B). Simultaneously, the expression of SOCS3 protein decreased in both mouse models of asthma compared with that in the control group (Figure 4C). Similar to the results of protein levels, the percent STAT3-positive area in the lung tissues of the OVA group and OVA + ozone group increased compared with that in the control group (Figures 4F,G), whereas the percent SOCS3-positive area decreased in the OVA + ozone group (Figures 4D,E). Single ozone exposure showed no significant statistical difference from the control group (Supplementary Figure S5).

### Expression of STAT3 and SOCS3 Was Correlated With Expression of Th17-Related Cytokines

**FIGURE 5:**
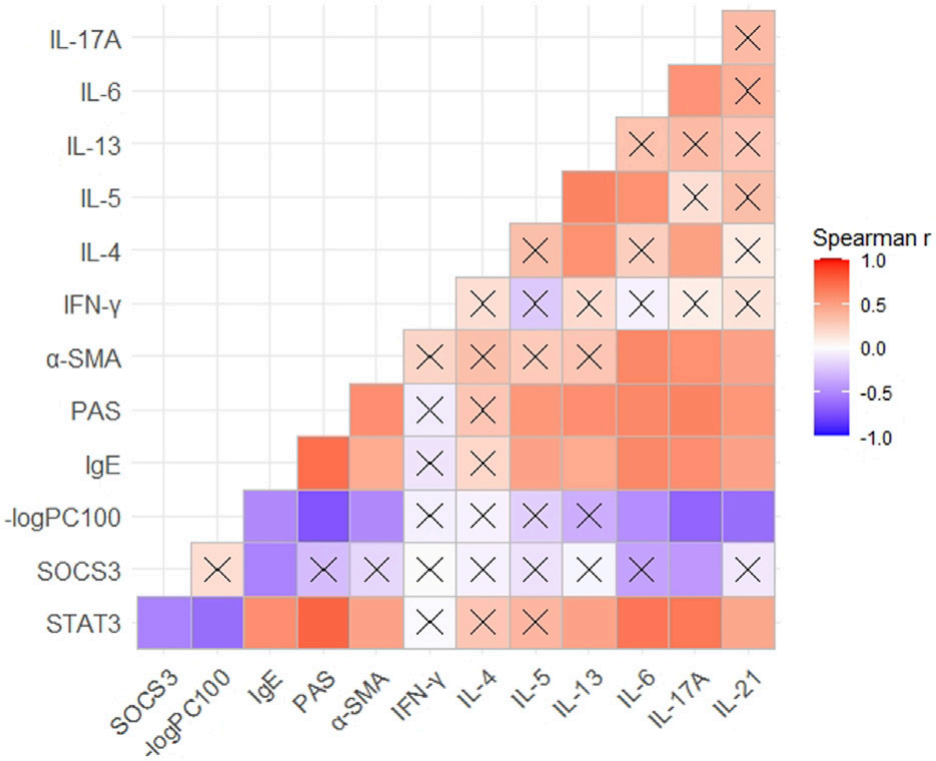
Correlation between the expression of STAT3 or SOCS3 and mucus secretion as well as inflammation, responsiveness, and remodeling of the airways in different asthma models. Spearman’s correlation coefficient matrix and Spearman’s rank correlation tests were conducted. The expression of STAT3, SOCS3, and α-SMA was discovered from the results of immunohistochemical analyses. The expression of IgE and Th1/Th2/Th17-related cytokines was obtained using ELISAs of serum. A cross indicates no significance. *p*-Values are shown in Supplementary Table S1. SOCS3, suppressor of cytokine signaling 3; STAT3, signal transducer and activator of transcription 3; α-SMA, α-smooth muscle actin.

Correlation was evaluated using Spearman’s rank analysis. STAT3 expression and SOCS3 expression showed a strong correlation with each other (r = −0.53). The expression of STAT3 and SOCS3 was correlated with the expression of IgE and IL-17A in mouse serum (r = 0.57 and 0.67 with STAT3 and −0.53 and −0.43 with SOCS3, respectively) (Figure 5). STAT3 expression showed a stronger correlation with the expression of Th17-related cytokines (IL-6, IL-17A, and IL-21) than Th2-related cytokines, in serum. STAT3 expression correlated with the percent stained areas of PAS and α-SMA (r = 0.75 and 0.46, respectively) and −logPC100 (r = −0.62) (Figure 5). Hence, STAT3 may be involved in the hypersecretion, remodeling, and hyperresponsiveness of airways observed in asthma.

### STAT3 and IL-6 Are Involved in the Corticosteroid Resistance of an Asthma Model Induced by OVA and Ozone

**FIGURE 6:**
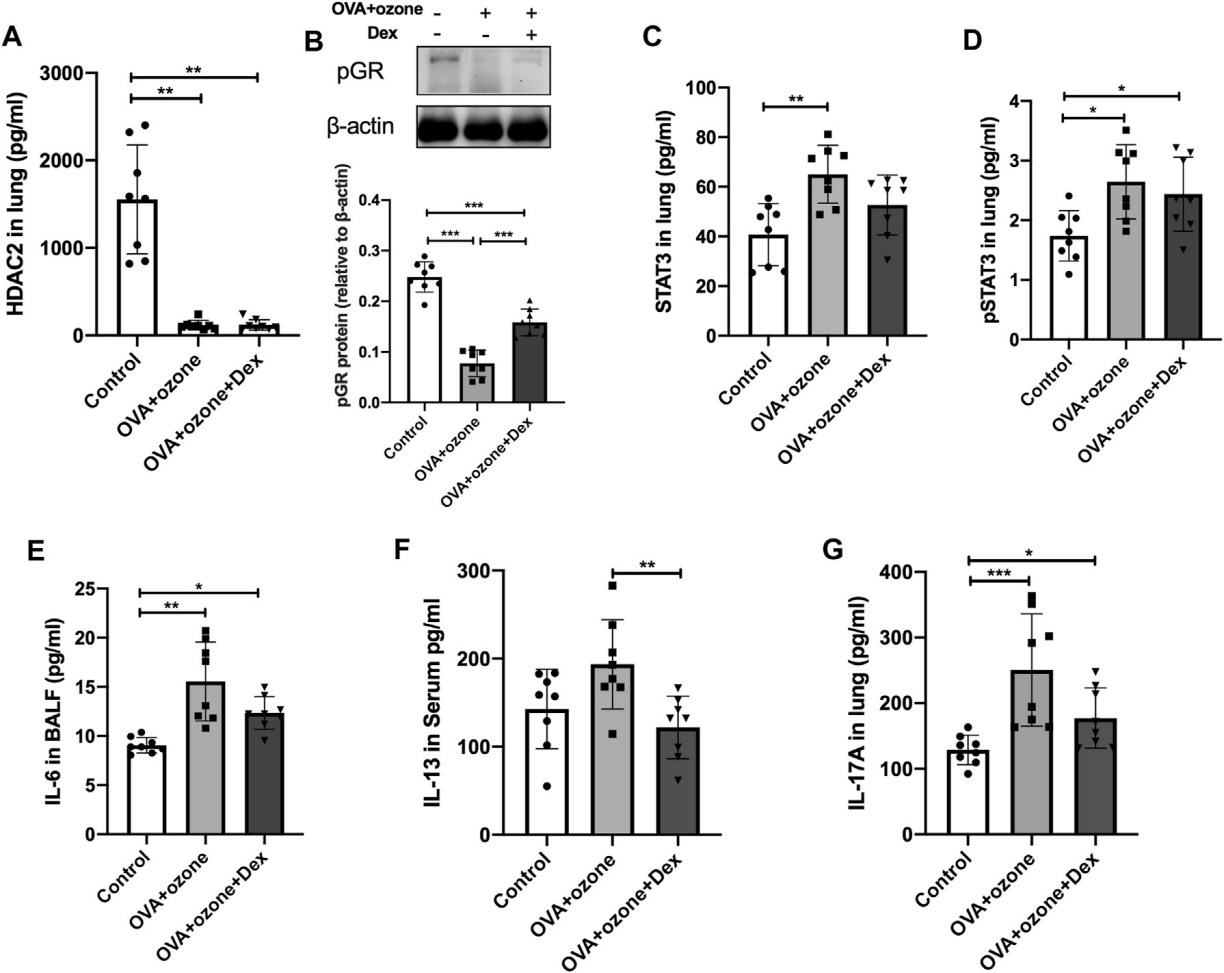
Changes in the expression of cytokines and transcription factors after intervention by dexamethasone. The expression of HDAC2, IL-6, IL-13, and IL-17A was measured by ELISAs. The expression of p-GR protein was analyzed by western blotting. **(A)** Expression of HDAC2 protein in lung tissue after Dex treatment. **(B)** Expression of p-GR protein in lung tissue after Dex treatment. **(C)** Expression of STAT3 protein in lung tissue after Dex treatment. **(D)** Expression of p-STAT3 protein in lung tissue after Dex treatment. **(E)** Expression of IL-6 protein in BALF after Dex treatment. **(F)** Expression of IL-13 protein after Dex treatment. **(G)** Expression of IL-17A protein after Dex treatment. Dex, dexamethasone; HDAC2, histone deacetylase 2; p-GR, phosphorylated-GR; STAT3, signal transducer and activator of transcription 3.*p < 0.05; **p < 0.01; ***p < 0.001.

The expression of HDAC2 and p-GR protein in the OVA + ozone group could not be restored completely by Dex treatment (Figures 6A,B). Protein expression of total STAT3 and p-STAT3 could not be reversed completely by Dex treatment (Figures 6C,D). IL-6 (which is important for the differentiation of Th17 cells and may be upstream of STAT3) expression appeared to be corticosteroid-insensitive in mice in the OVA + ozone group (Figure 6E). Protein expression of IL-13 (Th2-related cytokine) showed an evident response to this corticosteroid (Figure 6F), whereas IL-17A seemed to decline after Dex treatment, but continued to remain higher than that in the control group (Figure 6G).

### Expression of STAT3 and SOCS3 Was Altered in the Lung Tissue of Asthma Patients and Showed Correlation With Expression of IL-17A Protein

**FIGURE 7:**
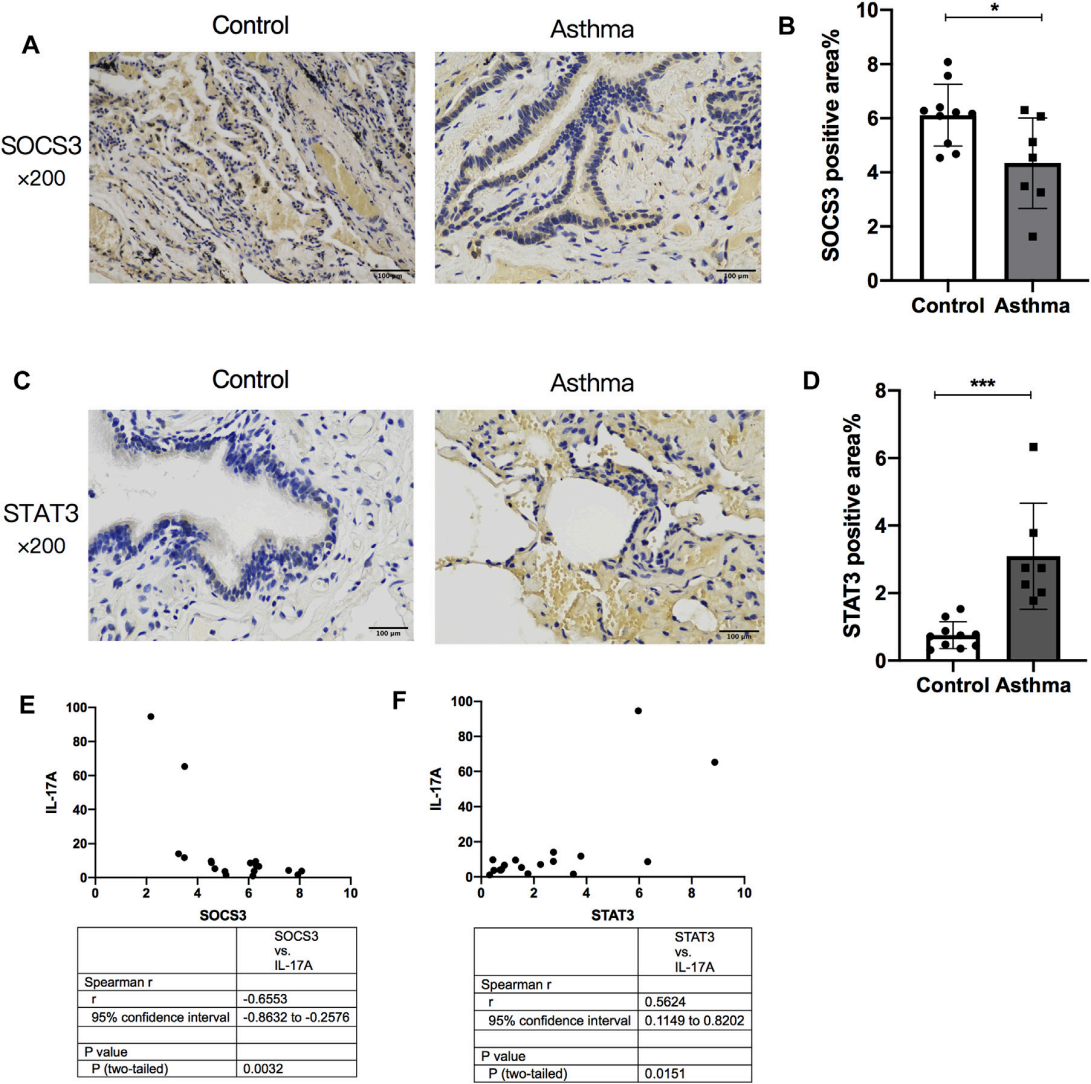
Comparison of SOCS3 expression and STAT3 expression in normal lung tissue between asthma patients and healthy controls and their correlation with IL-17A expression in the lung. The expression of STAT3 and SOCS3 in lung tissues of 17 patients undergoing thoracotomy for pulmonary nodules was analyzed by immunohistochemistry. **(A)** Representative photomicrographs of immunohistochemistry of SOCS3 in lung-tissue slices of the asthma group and control group. **(B)** Percent SOCS3-positive area around airways. **(C)** Representative photomicrographs of immunohistochemistry of STAT3 in lung-tissue slices of asthma and control groups. **(D)** Percent STAT3-positive area around airways. **(E)** Correlation between the percent STAT3-positive area and the expression of IL-17A protein in human lung tissue. **(F)** Correlation between the percent SOCS3-positive area and the expression of IL-17A protein in human lung tissue. SOCS3, suppressor of cytokine signaling 3; STAT3, signal transducer and activator of transcription 3. *p < 0.05; **p < 0.01; ***p < 0.001. Scale bar = 100 μm.

The normal lung tissues of seven asthma patients and 10 HCs who underwent thoracotomy were evaluated through IHC staining and ELISAs. The STAT3-positive area increased in the lung tissue of asthma patients compared with that in HCs (Figures 7A,B), whereas SOCS3 expression decreased in the lung tissue of asthma patients (Figures 7C,D). The expression of STAT3 and SOCS3 showed a strong correlation with the expression of IL-17A protein in the lung tissue of patients (r = 0.5624 and −0.6553, respectively) (Figures 7E,F). The characteristics of these patients at baseline are shown in Supplementary Table S2.

## Discussion

We established a T2-high asthma model with eosinophil infiltration and increased expression of Th2-related cytokines through OVA sensitization/challenge. We constructed a mixed Th2/Th17 response model with neutrophil infiltration and increased expression of Th17-related cytokines through OVA sensitization/challenge combined with ozone exposure.

The OVA + ozone group showed more severe airway inflammation, airway hyperresponsiveness, and severe airway remodeling than those in the OVA group. Th17-related cytokines have important roles in T2-low asthma (Chung, 2016; Svenningsen and Nair, 2017). To ascertain the differences in endotypes of our mouse models, we adopted cytokine antibody arrays for simultaneous and accurate detection of multiple cytokines.

After establishing models of T2-high asthma or a mixed Th2/Th17 response with neutrophil infiltration–based airway inflammation, we discovered the characteristics of corticosteroid resistance in the OVA + ozone group. Studies using mouse models of asthma and human type II alveolar epithelial cells have shown that exposure to high ozone levels can reduce the response of asthma patients to glucocorticoid therapy during acute exacerbations (Flayer et al., 2019). In our previous study, a mouse model induced by OVA and ozone showed the traits of corticosteroid resistance (Zhang J.-h. et al., 2019). In the present study, we demonstrated that the expression of HDAC2 and p-GR decreased in the OVA + ozone group, which is a characteristic of corticosteroid resistance. The development of corticosteroid resistance is closely related to the expression and phosphorylation of GRs. Reduced phosphorylation of GRs leads to reduced nuclear translocation, which results in corticosteroid resistance (Irusen et al., 2002). The anti-inflammatory effect of GRs is promoted by HDAC2, and studies have revealed that HDAC2 expression is decreased in patients with severe asthma and may be involved in the development of glucocorticoid resistance (Irvin et al., 2014). The expression of *Nr3c1* (the gene of GRs) (Farrell et al., 2018) was found to be increased, and synchronously, the expression of GR protein increased, in the OVA + ozone group. However, a reduction of expression of the active form of GR (p-GR), which may be facilitated by HDAC2, could have led directly to corticosteroid resistance in the OVA + ozone group.

As important pathophysiological features of severe asthma, neutrophil infiltration and activation in the lung are mostly mediated by CXCL8 and CXCL1 via CXCR1 and CXCR2 (Planagumà et al., 2015). Bao, A. et al. once found increased CXCL1 mRNA expression in an ozone-exposed ovalbumin mouse model (Bao et al., 2017). Johnston, R. A. and others showed an essential role of CXCR2 in maximal neutrophil recruitment after an acute O(3) exposure (Johnston et al., 2005). The ligand CXCL1 increased in our OVA + ozone–induced mouse model with neutrophil infiltration, indicating a role of chemoattractant for neutrophils in the steroid resistance of this model. The receptor CXCR2 showed a trend of increase compared with that in the control group while having no statistical difference. The difference in the mouse model and concentration or duration of ozone exposure may lead to the discrepancy of our results and Johnston, R. A. and others’. Further quantitative research should be conducted to determine the change of chemoattractant for neutrophils in the OVA + ozone mouse model.

Ozone can induce tissue injury due to oxidative stress. Some studies have shown that the hypoxia inducer HIF-1α is associated with oxidative stress and may affect asthma pathogenesis (Saini et al., 2010). HIF-1α expression in the OVA + ozone group showed an increasing trend compared with that in the control group, but the difference was not significant, which may have been related to the low ozone concentration (2 ppm) used in our modeling process. This finding suggested that oxidative-stress injury may not have been the leading cause of corticosteroid insensitivity in the OVA + ozone group.

Because of high expression of Th17-related cytokines (IL-6, IL-17A, and IL-21) in the OVA + ozone group, the expression of STAT3 and SOCS3 (which are important transcription factors of Th17) was evaluated further. The role of STAT3 and SOCS3 in asthma has been discussed in relation to the T2-mediated allergic response (Seki et al., 2003; Gao and Ward, 2007). Several studies have shown that short-term ozone exposure increased levels of STAT3 or p-STAT3 (Lang et al., 2008; Mishra et al., 2016a), while our study showed an increasing trend but no statistical difference between the ozone group and the control group. Maybe, the difference in methods and the duration of ozone exposure led to this discrepancy. There is still lack of study in effects of ozone exposure on SOCS3, and few scholars have focused on the role of STAT3 and SOCS3 in the mechanism of action of T2-low asthma and corticosteroid resistance. Inhibition of STAT3 expression by C188-9 was shown to reduce airway inflammation and airway remodeling in a house dust mite–induced asthma model created by Gavino and coworkers (Gavino et al., 2016). They adopted a different type of modeling compared with our model. However, studies have demonstrated the ability of house dust mites to induce the production of Th2 cells and Th17 cells simultaneously (Lan et al., 2011), which is similar to the model induced by OVA + ozone in our study. STAT3 expression increased in the OVA + ozone group. p-STAT3 expression increased in the OVA + ozone group more than that in the OVA group. Therefore, STAT3 activated after phosphorylation may have an important role in T2-low asthma and be involved in the development of corticosteroid resistance.

The transcription suppressor SOCS3 showed an opposite trend to that of STAT3 in the OVA + ozone group. This trend has been documented in other studies related to airway inflammation. Sun and others demonstrated the inhibitory effect of SOCS3 on differentiation of Th17 cells in an asthma model (Sun et al., 2018). However, Mishra and coworkers discovered different results: the expression of SOCS3 and STAT3 increased synchronously in allergic airway inflammation (Mishra et al., 2016b). The reason for this disparity may be related to differences in the mouse model employed. Those controversial results further demonstrate the complex regulatory role of SOCS3 in asthma pathogenesis. Some studies have discussed the role of STAT3 in corticosteroid resistance (Banuelos et al., 2016; Halwani et al., 2016). However, few studies have paid attention to the potential role of SOCS3 in corticosteroid resistance in an asthma model closely related to Th17 cells.

We showed that the expression of IL-6 and IL-17A was correlated with that of STAT3 and SOCS3. STAT3 showed a stronger correlation with the expression of Th17-related cytokines in serum (e.g., IL-6 and IL-17A) than the expression of Th2-related cytokines, which indicates its interaction with Th17 cells. The correlation between the expression of STAT3 or SOCS3 and airway inflammation indicated that STAT3 and SOCS3 are involved in asthma. A strong correlation between SOCS3 expression and asthma severity and serum IgE levels in allergic patients has been reported (Gao and Ward, 2007), which is in accordance with our findings in mouse models. Gavino and others found that blockade of Th2 and Th17 pathways by STAT3 inhibitors in a mouse model of asthma was effective in reducing lung inflammation compared with that employing blockade of one pathway alone (Gavino et al., 2016), which conformed to the correlation between STAT3 expression and IL-13 and IL-17A expressions in our study. However, we speculate that STAT3 expression may be correlated more closely with Th17 pathways than Th2 pathways in the present study due to its stronger correlation with Th17-related cytokines rather than Th2-related cytokines.

As demonstrated above, STAT3 expression is correlated with mucus secretion as well as inflammation, responsiveness, and remodeling of the airways, which suggests an important role of STAT3 in asthma pathogenesis. We found that STAT3 phosphorylation as well as the expression of IL-6 and IL-17A could not be reversed by Dex treatment, which indicates their involvement in corticosteroid resistance of OVA- and ozone-induced asthma. In an animal study, Banuelos and collaborators showed that IL-17A production was not suppressed by glucocorticoids (Banuelos et al., 2016). Previously, we found that treatment with the IL-17A monoclonal antibody reduced corticosteroid insensitivity in a mouse model of asthma (Zhang Y. et al., 2019). Here, we paid more attention to p-STAT3. It has been reported that STAT3 phosphorylation in Th17 cells is insensitive to glucocorticoid inhibition (Banuelos et al., 2016). IL-6 is involved in STAT3-regulated inflammation and may interact with STAT3 (Rottenberg and Carow, 2014). Dex and budesonide help protect human type II alveolar epithelial cells by epithelium-derived surfactant protein D in a manner that is dependent on GRs and STAT3 (an IL-6–responsive transcription factor) (Flayer et al., 2019). Thus, IL-6 and STAT3 may have important roles in an OVA- and ozone-induced corticosteroid-resistant asthma model. Our analyses in the human lung tissue of the expression of STAT3 and SOCS3 further convinced us that STAT3 and SOCS3 are involved in asthma. We analyzed the correlation between the expression of STAT3 or SOCS3 and IL-17A. The expression of IL-17A in the lung tissue of asthma patients and HCs showed a strong correlation with the expression of STAT3 and SOCS3. These findings further indicated the possible role of interaction between STAT3 or SOCS3 and Th17 cells in asthma. The expression of SOCS3 and STAT3 has been evaluated in airway inflammation in some animal models (Gao and Ward, 2007; Santana et al., 2019; Pinheiro et al., 2020), but we are the first to observe their changes in the lung tissue of asthma patients. Though we failed to divide asthma patients to T2-high or -low endotypes, eosinophil counts in blood were <150/µl in the asthma group and did not show a significant difference from that in the control group (Supplementary Table S2).

However, there are several limitations of the current study. Firstly, increasing studies have demonstrated non-HLA antibodies such as Col-V and EGFR involved in the IL-17–related pathways in different pulmonary diseases (and may contribute to severe asthma). More attention should be paid to these pathways but not the conventional pathway. We will try to involve these non-HLA antibodies in our studies of IL-17 and its pathways. Secondly, further studies on the intervention of STAT3 and SOCS3, as well as the discussion of the mechanisms of their regulation in Th17 cells and corticosteroid resistance, should be conducted.

## Conclusion

We established an asthma model with neutrophil infiltration and increased expression of Th17-related cytokines by OVA sensitization/challenge and ozone exposure. The OVA + ozone group showed corticosteroid resistance with reduction of expression of HDAC2 and p-GR. STAT3 and SOCS3 appeared to be involved in asthma pathogenesis in mouse models and asthma patients. IL-6 and STAT3 may have contributed to corticosteroid resistance in the OVA + ozone group through regulation of Th17 cells. SOCS3 (a negative transcription factor) may interact with STAT3 and be involved in the corticosteroid insensitivity of the T2-low asthma model.

## Data Availability

The raw data supporting the conclusions of this article will be made available by the authors, without undue reservation.

## Abbreviations

ACh, acetylcholine chloride; α-SMA, α-smooth muscle actin; BALF, bronchoalveolar lavage fluid; CXCL1, chemokine (C-X-C motif) ligand 1; CXCR2, chemokine (C-X-C motif) receptor 2; Dex, dexamethasone; ELISA, enzyme-linked immunosorbent assay; FEV1, forced expiratory volume in one second; FVC, forced vital capacity; GR, glucocorticoid receptor; HDAC2, histone deacetylase 2; H&E, hematoxylin and eosin; IHC, immunohistochemical; OVA, ovalbumin; PAS, periodic acid-Schiff; PC100, the ACh concentration required to increase R_L_ by 100% from baseline; p-GR, phosphorylated-GR; p-STAT3, phosphorylated-STAT3; R_L_, lung resistance; SOCS3, suppressor of cytokine signaling 3; STAT3, signal transducer and activator of transcription 3.
