# Supplementary material for: STAT3 and IL-6 Contribute to Corticosteroid Resistance in an OVA and Ozone-induced Asthma Model with Neutrophil Infiltration
Source: Front Mol Biosci. 2021 Oct 25;8:717962. doi: 10.3389/fmolb.2021.717962 (PMC8573338; doi:10.3389/fmolb.2021.717962)

**Table S1- *P value of correlation analysis in Figure 5***

|  | STAT3 | SOCS3 | -logPC100 | IgE | PAS | α-SMA | IFN-γ | IL-4 | IL-5 | IL-13 | IL-6 | IL-17A |
| --- | --- | --- | --- | --- | --- | --- | --- | --- | --- | --- | --- | --- |
| STAT3 | 0 |  |  |  |  |  |  |  |  |  |  |  |
| SOCS3 | 0.0077 |  |  |  |  |  |  |  |  |  |  |  |
| -logPC100 | 0.0013 | 0.4221 |  |  |  |  |  |  |  |  |  |  |
| IgE | 0.0034 | 0.0076 | 0.0121 |  |  |  |  |  |  |  |  |  |
| PAS | 0.0000 | 0.1872 | 0.0001 | 0.0001 |  |  |  |  |  |  |  |  |
| α-SMA | 0.0229 | 0.4257 | 0.0129 | 0.0421 | 0.0035 |  |  |  |  |  |  |  |
| IFN-γ | 0.9180 | 0.9694 | 0.7666 | 0.6507 | 0.6936 | 0.3148 |  |  |  |  |  |  |
| IL-4 | 0.1755 | 0.8090 | 0.8114 | 0.3381 | 0.1723 | 0.1219 | 0.4647 |  |  |  |  |  |
| IL-5 | 0.0730 | 0.5797 | 0.3859 | 0.0221 | 0.0089 | 0.2280 | 0.2940 | 0.1184 |  |  |  |  |
| IL-13 | 0.0229 | 0.8353 | 0.1006 | 0.0372 | 0.0034 | 0.1644 | 0.3963 | 0.0052 | 0.0016 |  |  |  |
| IL-6 | 0.0003 | 0.0632 | 0.0169 | 0.0020 | 0.0022 | 0.0020 | 0.8235 | 0.2464 | 0.0054 | 0.1449 |  |  |
| IL-17A | 0.0004 | 0.0352 | 0.0004 | 0.0034 | 0.0014 | 0.0046 | 0.7098 | 0.0166 | 0.4440 | 0.0893 | 0.0068 |  |
| IL-21 | 0.0312 | 0.6876 | 0.0012 | 0.0204 | 0.0087 | 0.0198 | 0.5447 | 0.6566 | 0.1150 | 0.1684 | 0.0529 | 0.0906 |

**Table S2- *Demographic data in the control group and asthma group of human.***

| **Characteristics and variables** | **Control**  **(n = 10)** | **Asthma**  **(n = 7)** | **P value** |
| --- | --- | --- | --- |
| **Age (years)§** | 57.00±7.54 | 56.43±8.58 | 0.88 |
| **Gender, male (n,%)** | 6 (60.0%) | 3 (42.8%) | 0.64 |
| **Height (cm)§** | 164.20±7.61 | 161.70±4.82 | 0.423 |
| **Weight (kg)§** | 67.40±13.05 | 59.00±3.06 | 0.078 |
| **BMI (kg/m^2^)§** | 24.81±3.20 | 22.57±0.99 | 0.061 |
| **Fomer smoker (n,%)** | 2 (20.0%) | 1 (14.3%) | ＞0.999 |
| **FVC%pred§** | 96.15±10.17 | 89.92±7.44 | 0.137 |
| **FEV_1_%pred§** | 92.19±10.66 | 87.46±7.90 | 0.101 |
| **FENO (ppb)§** | 35.33±18.71 | 50.71±21.62 | 0.160 |
| **EOS in the blood (*10^9^/L)§** | 0.143±0.101 | 0.159±0.063 | 0.702 |
| **EOS% in the blood (%)§** | 3.04±2.12 | 3.81±1.43 | 0.382 |
| **Size of the lung nodule (mm)** | 11.90±7.23 | 10.29±4.99 | 0.594 |
| **Pathological type (adenocarcinoma/ epidermoid carcinoma/benign tumor)** | 6/0/1 | 6/0/4 | \ |

§ Mean±SD values.

**Figure S1- *Statistical analyzed figure of cytokines（except for IL-6, IL-17A, IL-21) in Figure 1A***

***
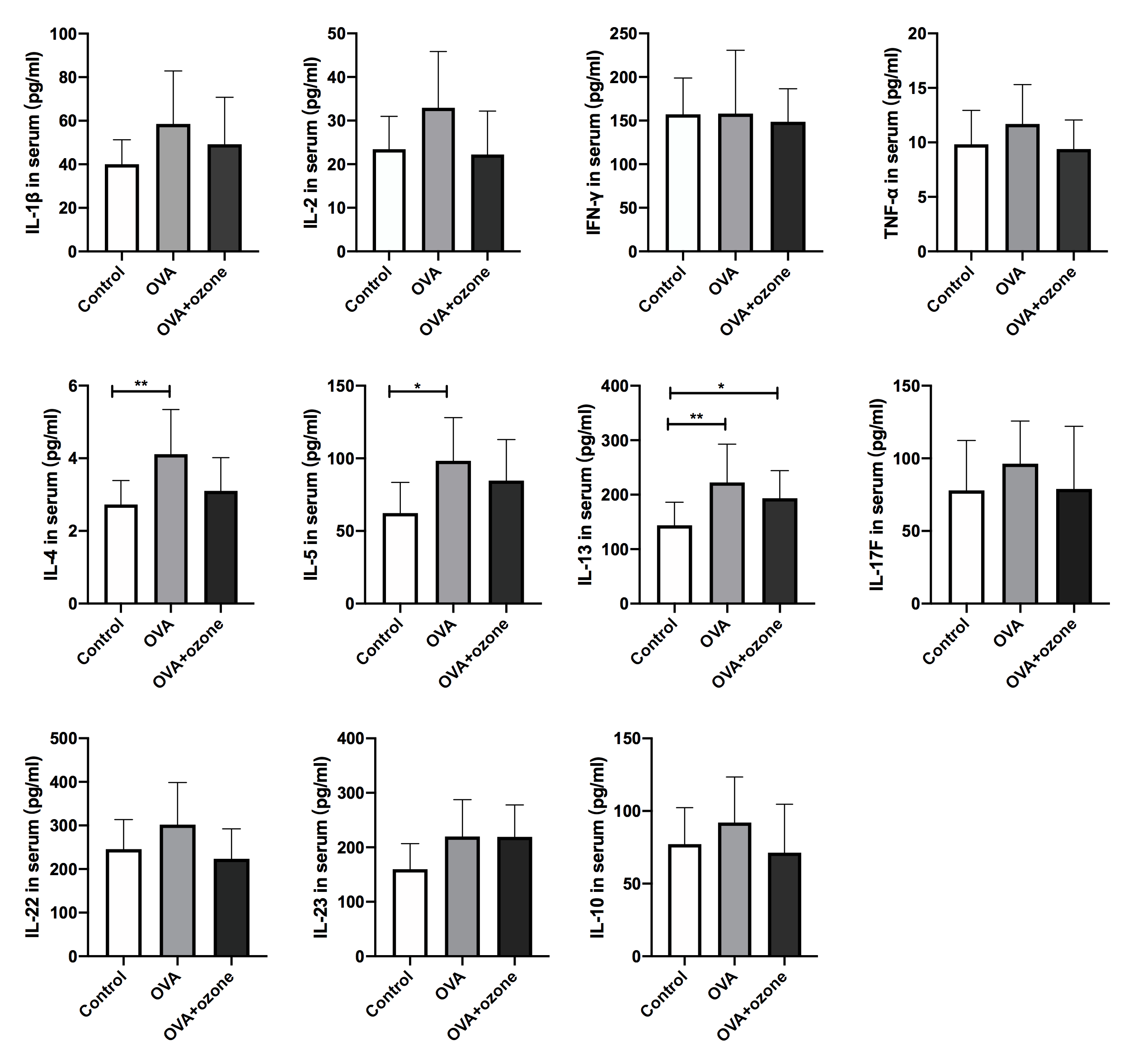
***

**Figure S2- *Levels of Th2-related cytokines in BALF***

***
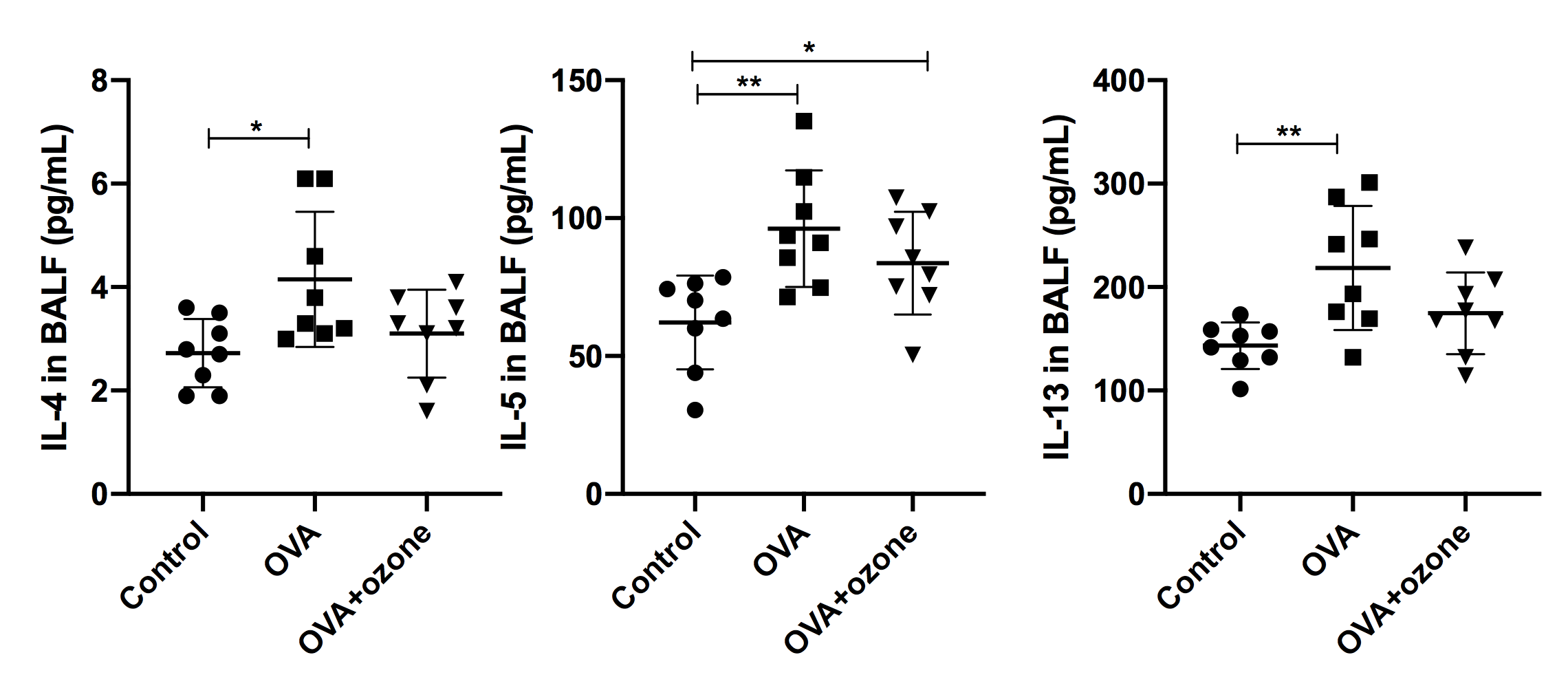
***

IL-4, IL-5 and IL-13 levels in BALF were measured by ELISA.

**Figure S3- *IHC of CXCR2 in lung of mice.***

***
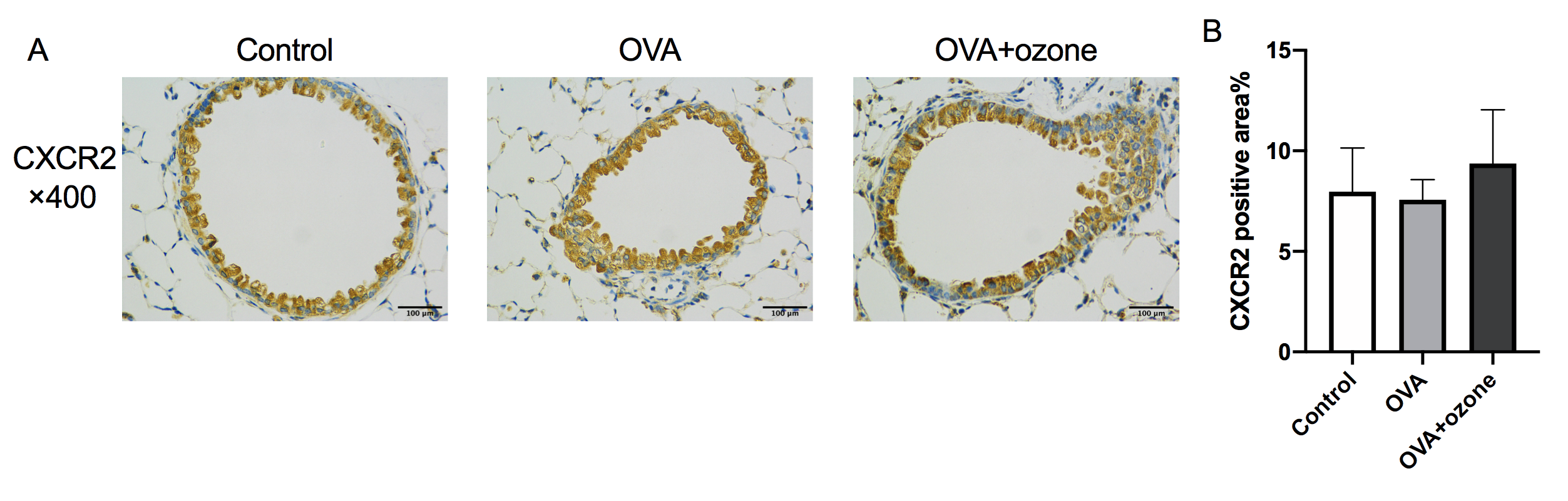
***

1. Representative photomicrographs of immunohistochemistry of CXCR2 in lung-tissue slices.

(B) Percent CXCR2-positive area around airways.

**Figure S4- *Airway inflammation and responsiveness of the ozone group.***


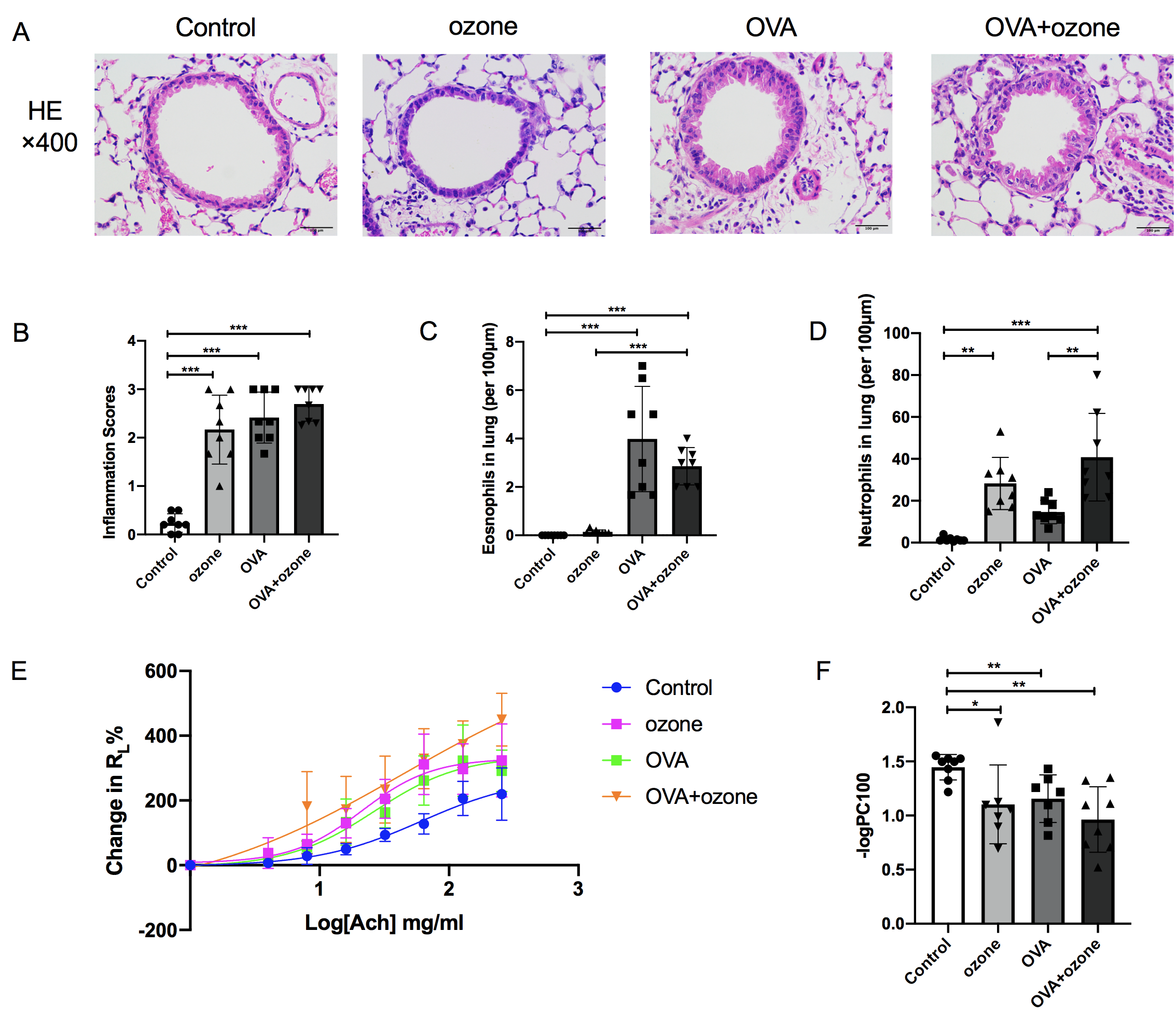


1. Representative photomicrographs of lung with inflammatory-cell infiltration and hyperemia in hematoxylin and eosin-stained sections.
2. Airway inflammation scores.

(C) Density of neutrophil infiltration.

(D) Density of eosinophil infiltration.

(E) Mean percent increase in R_L_ in response to increasing concentrations of ACh.

(F) –logPC100 (the ACh concentration required to increase R_L_ by 100% from baseline).

**Figure S5- *Expression of STAT3 and SOCS3 mRNA in lung tissue of ozone group.***


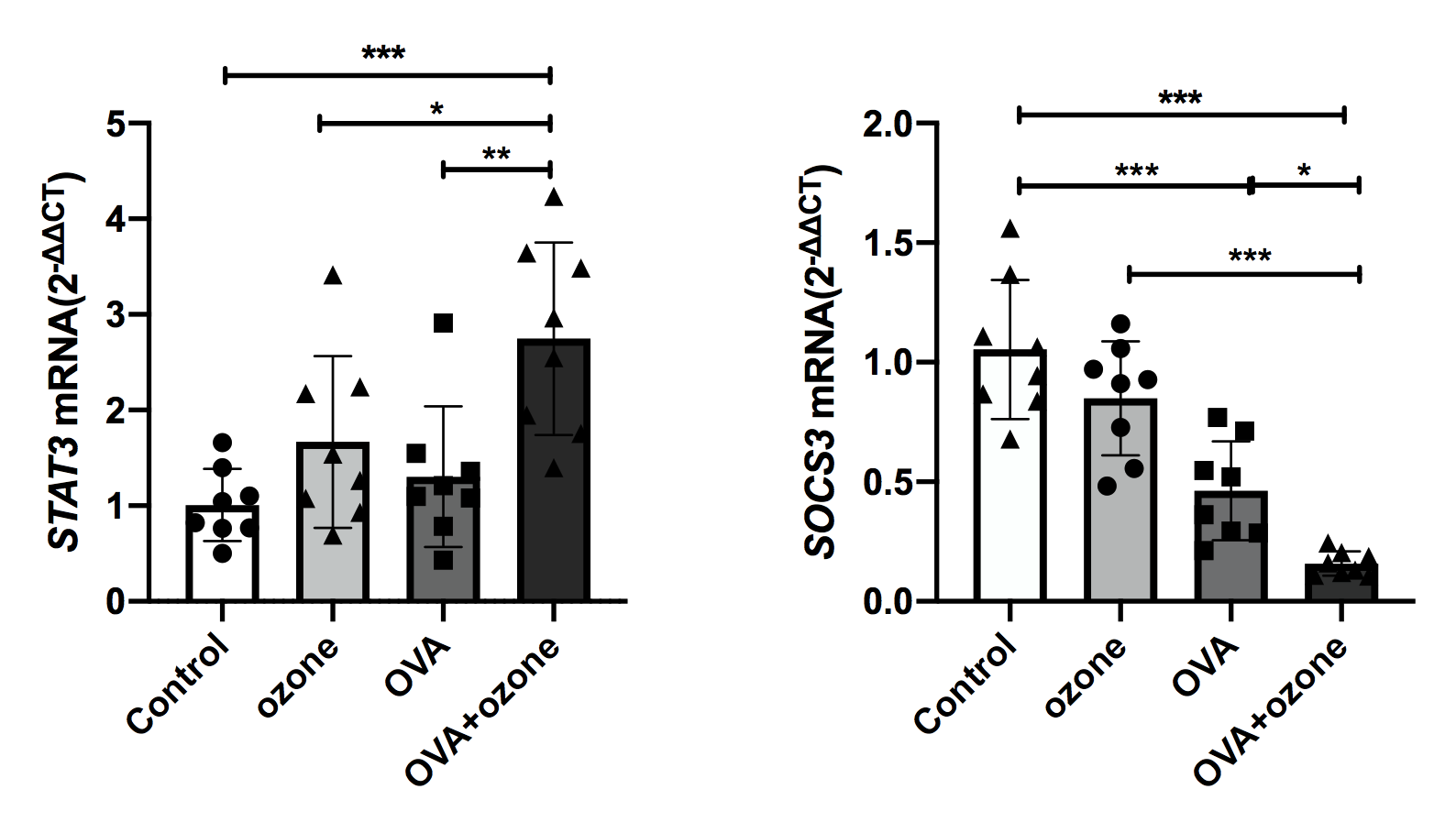

Supplement: Supplementary file 1 [file DataSheet1.docx]
